# Ultrathin Gold Nanowires

**DOI:** 10.3390/nano15060428

**Published:** 2025-03-11

**Authors:** Shuo Liu, Chunmeng Liu, Ye Wang, Jiaqi Zhang, Shaobo Cheng, Chongxin Shan

**Affiliations:** 1 Henan Key Laboratory of Diamond Optoelectronic Materials and Devices, Key Laboratory of Materials Physics, Ministry of Education, School of Physics, Zhengzhou University, Zhengzhou 450052, China; liushuoxyz@163.com (S.L.); chunmeng@zzu.edu.cn (C.L.); cxshan@zzu.edu.cn (C.S.); 2 Center of Advanced Analysis and Gene Sequencing, Zhengzhou University, Zhengzhou 450001, China; 3 Key Laboratory of Material Physics, Ministry of Education, School of Physics and Microelectronics, Zhengzhou University, Zhengzhou 450052, China; 4 Institute of Quantum Materials and Physics, Henan Academy of Sciences, Zhengzhou 450046, China

**Keywords:** Au nanowires, structural stability, Young’s modulus, density functional theory

## Abstract

Nanowires (NWs), particularly Au NWs, have garnered significant attention for their exceptional properties and applications as nanoscale interconnects in micro-nano electronics. Nevertheless, the stable structure of sub-2nm Au NWs continues to be ambiguous due to the significant challenges in both the fabrication processes and direct atomic-scale structural characterization. This study employs in situ transmission electron microscopy (TEM) techniques combined with the Perdew–Burke–Ernzerhof (PBE) functional within density functional theory (DFT) to systematically investigate the intrinsic relationship between the atomic structure and stability of oriented Au NWs. Our results indicate that the structural stability of Au NWs is influenced by both their structural symmetry and the proportion of (111) surfaces. Additionally, the Young’s modulus of Au NWs is related to their cross-sectional symmetry, with an inverse correlation observed when the equivalent radius is below 6 Å. Finally, the number of conductive channels in Au NWs increases with cross-sectional size, with higher symmetry exhibiting more conducting channels. The experimental results offer significant insights into the key determinants influencing the structural integrity of ultrathin gold nanowires, which serves as a crucial basis for their implementation in next-generation nanoscale device technologies.

## 1. Introduction

Au nanowires (NWs) play a crucial role in nanodevices due to their exceptional physical and chemical properties, including high antioxidation resistance and excellent electrical conductivity. They also serve as the primary material for the electrical connections in these devices [1,2,3,4]. Given the remarkable potential of Au NWs for diverse applications, significant research efforts have been devoted to investigating their fundamental properties and functional performance [5,6]. Nanoscale materials often exhibit unique physical and chemical properties [7,8]. For example, previous experimental results have shown that the Young’s modulus and electrical properties of Au NWs deviate from their bulk values as the size is reduced, exhibiting size-dependent variations [9,10]. De Heer et al. demonstrated that metal NWs exhibit two stable diameter series that compete with each other, a phenomenon influenced by atomic and electronic shell-filling effects [11]. Moreover, due to the size dependence of NWs, the classical microscopic Ohm’s law is no longer applicable to the electron transport in independent NWs. Specifically, for free-standing Au NWs with diameters less than 2 nm, their electrical properties are primarily characterized by ballistic conduction and quantized conductance ($G\approx NG_{0}$, *N* is the number of conductance channels and ${\;G}_{0}=2e^{2}/h$, where *e* represents the electron charge, and *h* denotes the Planck constant) [10,12,13]. However, when nanowires or atomic chains are embedded or in some certain environments, these conditions can influence the tunneling phenomenon, leading to variations in conductance across different environments [14,15]. For example, previous experiments have shown that the conductance of a gold monoatomic chain is 1$G_{0}$, while that of a diatomic chain is 2$G_{0}$ [16]. Moreover, experimental observations frequently reveal that NWs with certain conductance values occur more often, likely corresponding to the stable structural configurations of the NWs [9,17,18,19]. Regarding the synthesis of gold nanowires, researchers have conducted a series of in-depth studies.

Regarding the synthesis and stability of Au NWs, researchers have conducted extensive and in-depth studies from both experimental and computational perspectives. The team of Z. Mamiyev demonstrated through one-dimensional dispersive plasmon excitation experiments that even single-atom gold wires can be successfully prepared and studied in experimental conductivity. Although one-dimensional wires exhibit instability at finite temperatures, the hybridization of silicon surface states with adsorbates can lead to the formation of stable dimerized double gold chains. This suggests that doping and/or charge corrections on gold quantum chains can effectively enhance their stability [20,21,22,23,24].

However, the structural stability of Au NWs, which is closely related to their fabrication, performance, and service life, has not been thoroughly investigated. While Au has long been recognized as a conventional material exhibiting remarkable physical and chemical properties, the manifestation of novel physicochemical characteristics becomes evident as its dimensionality is reduced to the nanoscale [25]. For instance, unique structures, such as helical multi-shell Au NWs and ultra-narrow Au nanoribbons, have been experimentally observed using high-resolution transmission electron microscopy (HRTEM) [26]. It has also been noted that there is a correlation between the cross-sectional structure and the stability of the NWs. For example, Kurui et al. demonstrated that oriented Au NWs are more stable when they adopt a hexagonal prism structure [27]. In addition, metal nanowires fabricated through nanotechnology have a wide range of direct practical applications. For example, silicon NWs are widely used in devices such as lithium-ion batteries, semiconductor nanowires are commonly employed in the fabrication of photodetectors, and metal NWs such as Au and silver play a significant role in the thermophotovoltaic response of nanowire networks [25,28,29].

Despite extensive experimental studies having been conducted on Au NWs, certain fine structures remain challenging to precisely capture and characterize due to their ultra-small dimensions with diameters less than 2 nm. Moreover, their cross-sectional structure cannot be directly observed, as the HRTEM images obtained in the experiment are two-dimensional projections of the Au NWs. Therefore, there is a lack of accurate characterization of the cross-sectional structure, and the symmetry of Au NWs remains enigmatic. In addition, it remains unclear whether there is a correlation between the Young’s modulus of Au NWs, the number of conductive transport channels, and their cross-sectional configuration. Simulation calculations play a significant role in complementing and refining the experimental results, making them more comprehensive and clear. In this study, the use of density functional theory (DFT) not only provides theoretical support for experimental hypotheses but also establishes a solid foundation for the analysis and interpretation of the experimental results [30,31,32,33]. Furthermore, they can predict unknown performance, which is essential for advancing nano-electromechanical systems (NEMSs) and promoting the widespread application of NWs, such as enabling the accurate 3D visualization of their cross-sections.

In this study, we investigated the impact of cross-sectional structures on the stability, Young’s modulus, and electrical properties of oriented Au NWs with diameters smaller than 1 nm. This was achieved using in situ TEM combined with first-principles DFT calculations. Our findings establish a correlation between cross-sectional symmetry, equivalent radius, the proportion of densely packed (111) surfaces, and the properties of Au NWs. Additionally, we provide insights into the broader applicability of our results.

## 2. Methodology

### 2.1. In Situ TEM Experiment

**Figure 1:**
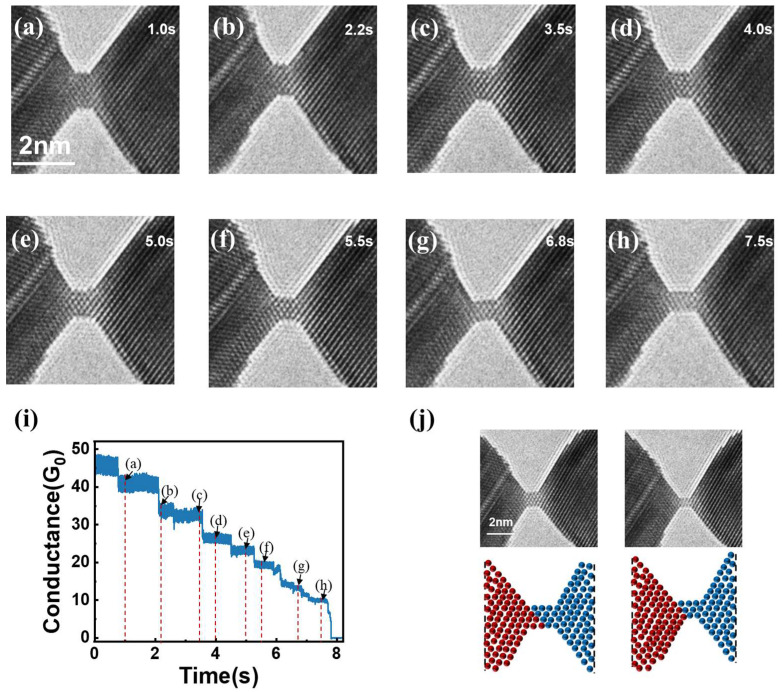
(**a**–**h**) are the TEM images corresponding to the stretching process shown in Figure (**i**), (**i**) is the conductance curve during the stretching process, (**j**) shows the TEM images and atomic structure model corresponding to the stretching deformation process of the <110> oriented contact. The boundary between the blue and red atoms represents the slip plane.

We conducted experiments using the JEM-2000FXV (JEOL, Tokyo, Japan) transmission electron microscope (TEM) under ultra-high vacuum conditions (10^−7^ Pa). In the TEM, two gold wires (99.99%) were controlled to make contact using our home-developed in situ TEM holder [10,34]. The positioning of the Au wire was precisely regulated through a dual-stage manipulation system, employing a piezoelectric tube actuator for nanometer-scale fine adjustments and an ultrasonic motor for micrometer-range coarse positioning. The Au wires were baked at ~130 °C for at least 24 h in a vacuum chamber before the experiment. Then, the Au wires were irradiated with a strong electron beam under a beam density of ~100 A/cm^2^ in the TEM column to remove the contaminates and gas adsorbed on the wires. The electrical conductance of the Au nanocontacts (NCs) was evaluated by applying a 10 mV bias voltage to the NCs, followed by the acquisition and amplification of the voltage signal through a current amplifier. The TEM images and conductance curves during the stretching process are shown in Figure 1.

### 2.2. First-Principle Calculations

To investigate which cross-sectional configuration of the nanowires has more stable mechanical properties, we conducted a simulation study. DFT was employed to investigate the structural stability, electronic properties, and mechanical properties of Au NWs [35,36,37]. The <110> oriented Au NWs were chosen because this orientation is the most commonly observed in experiments [38,39]. All the computational simulations were conducted utilizing the DMol^3^ code, in which the physical wave functions are expanded within an accurate numerical basis set specifically optimized for cluster calculations [40]. In order to make the calculation more accurate and concise, a double-zeta, atom-centered basis set (DND) [41] and generalized gradient approximation with the Perdew–Burke–Ernzerhof (GGA-PBE), along with a hardness conserving semilocal pseudopotential, were used to deal with the exchange-correlation potential in the computational process, which has been widely used to explore the physical properties of the materials [42,43,44]. The size of the DND basis is comparable to Gaussian 6-31G* but much more accurate. Furthermore, our calculations also take into account the effects of spin polarization. The conjugate gradient method based on the delocalized internal scheme was used to optimize the geometry of the cross-section of the Au NWs [45,46]. The computation is considered complete when the maximum total displacement fluctuation is within 0.005 Å and the energy fluctuation is within 1 × 10^−5^ Ha.

## 3. Results and Discussion

Through in situ TEM observations, the formation of NCs was observed during the gradual approach of two gold nanowires. The subsequent elongation process revealed a progressive thinning of these NCs, with the corresponding structural evolution and electrical transport characteristics documented in Figure 1a–h and Figure 1i, respectively. From the conductance curve, it is evident that the conductance decreases in a stepwise manner as the stretching progresses. This behavior suggests that the deformation of the contact is a periodic combination of elastic and plastic deformation. The conductance plateau corresponds to elastic deformation, while the sudden drops in conductance indicate plastic deformation. By correlating the conductance curve with TEM images, we observe that the contact structure remains unchanged during the plateau period (elastic deformation). However, when the conductance experiences a sudden drop, the contact structure undergoes a sharp narrowing, indicative of plastic deformation.

**Figure 2:**
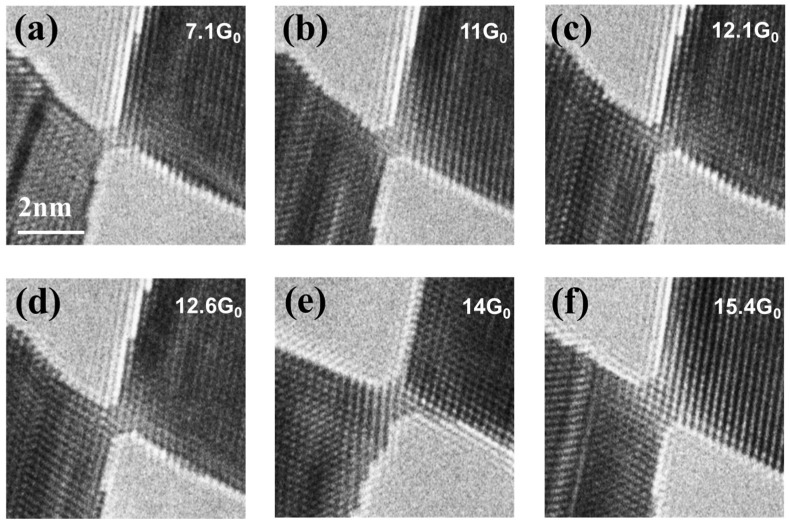
The structures that appeared most frequently in the experiment. (**a**–**f**) TEM images corresponding to the structures.

The conductance curve observed during stretching exhibits a behavior similar to that of Au <111> NCs reported in previous studies (stepwise decrease in conductance) [10]. Prior research has shown that Au <111> NCs undergo thinning through the sequential introduction of {111} layers perpendicular to the NCs. However, the deformation mechanism of Au <110> NCs follows a different pattern. A detailed analysis of the TEM images during stretching reveals that, in the case of Au <110> NCs, deformation occurs via the slip of the {111} plane along the <112> direction, as illustrated in Figure 1j. This distinction highlights a fundamental difference in the structural evolution of Au NCs along different crystallographic orientations. Furthermore, our experiments reveal that certain structures frequently appear, as shown in Figure 2a–f, suggesting that they may be relatively stable. Nevertheless, as TEM imaging is limited to two-dimensional projections, we complement our experimental observations with first-principles computational analyses to systematically investigate these recurrent structural configurations and elucidate the fundamental mechanisms governing their stability.

**Figure 3:**
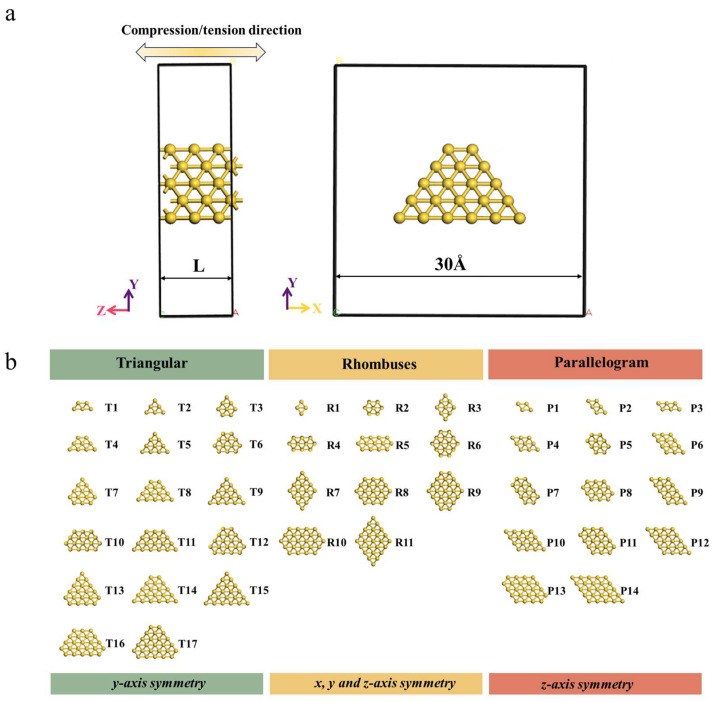
Schematic diagram of the theoretical model of Au NWs. (**a**) Front and side view of a built Au NW, (**b**) cross-sectional atomic arrangement of equilateral triangles (T), rhombuses (R), and parallelograms (P) series structures. Here, T, R, and P represent the basic unit shapes of the cross-sections as equilateral triangles, rhombuses, and parallelograms, respectively. Symmetry refers to the property that the cross-section can completely overlap with its original shape after a 180° rotation around a spatial coordinate axis. Taking *x*-axis symmetry as an example, if a cross-section possesses *x*-axis symmetry, it will completely coincide with its original shape after a 180° rotation around the *x*-axis. Additionally, we have numbered each type of cross-section based on the number of atoms in the cross-section of the NWs. For example, “R7” denotes the seventh type of cross-section in the R series NWs.

Figure 3a shows the front and side views of a constructed Au NW that extends infinitely along the direction (*z*-axis). The dimensional parameters of all the computational models were optimized to account for both first-nearest-neighbor interactions and long-range atomic correlations. To calculate the physical properties of Au NWs under tensile or compressive deformation, the model was placed in a unit cell with dimensions of 30 Å, 30 Å, and “L” along the x, y, and z directions, respectively. The large vacuum slab in the x and y directions effectively prevents spurious interactions between the Au NWs. To simulate the deformation process of Au NWs, uniaxial compression/tension along the z direction is applied by adjusting the L value (the length of the wire) near the equilibrium position in steps of 0.1 Å [47]. The oriented Au NWs with a radius ranging from approximately 2.31 Å to 5.76 Å were calculated, as shown in Figure 3b. The cross-sections of the oriented Au NWs were categorized into three types based on the symmetry of the individual sections: equilateral triangles (T, with *y*-axis symmetry), rhombuses (R, with *x*, *y*, and *z*-axis symmetry), and parallelograms (P, with *z*-axis symmetry). The three-dimensional symmetry with respect to the *x*, *y*, and *z* axes is defined by the condition that any cross-sectional plane within the coordinate system (illustrated in Figure 3a) remains invariant under 180° rotational transformations about each respective axis. The cross-section before and after rotation can perfectly coincide, as illustrated in Figure 3b. Since the cross-sections of actual NWs are not perfectly circular, we introduced the concept of “equivalent radius” ($R_{e}$) to simplify the description of NWs’ thickness and minimize the influence of shape on the results. The equivalent radius is defined as follows [48]:

(1) Re=NLV∕π12

where *N* is the number of atoms in the unit cell, *L* is the unit cell length, and *V* is the atomic volume of Au. Since the size of the Au NWs which we studied is smaller than the Fermi wavelength of electrons, they exhibit the characteristic of quantized conductance. The number of conductance channels of these Au NWs is determined by the number of bands that pass through the Fermi level in the calculated band structure. When the cross-section of the wire increases beyond a certain point, the quantum conduction mechanism is inevitably disrupted due to factors such as surface scattering. As a result, the actual conductance may be relatively lower than the number of conductive pathways [49]. Building on the above, the charge density profiles of Au NWs with different cross-sections in the T, R, and P series were calculated.

**Figure 4:**
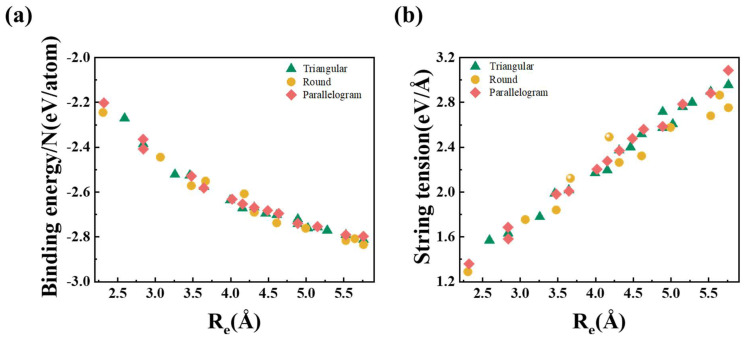
Stability-related calculations for the T, R, and P series. (**a**) The local minimum binding energy and (**b**) string tension as a function of equivalent radius ($R_{e}$).

The calculated energy, Young’s modulus, and electrical properties of Au NWs with three different cross-sectional structures (T, R, and P) are summarized in Table 1. To further elucidate the influence of the cross-sectional structure on the stability of Au NWs, a detailed discussion of these results is provided later. Figure 4a shows the calculated binding energy as a function of the $R_{e}$ of Au NWs. The binding energy is quantitatively defined as the energy difference between an individual atom within a fully optimized Au NW configuration and its corresponding isolated Au atom in the ground state. For all Au NWs with T, R, and P configurations, the absolute value of binding energy increased with the $R_{e}$, and there is no significant difference between the three systems. The results indicate that the atomic configurations of the NWs have a limited influence on their binding energy, assuming an ideal NW with infinite length.

However, in practice, Au NWs are typically fabricated through a contact-stretching process involving two gold tips. As a result, the length of the Au NW is finite and influenced by the two gold tips at both ends. Owing to their instability, ultrathin NWs exhibit a propensity to undergo structural relaxation into energetically favorable configurations. Therefore, ultrathin NWs inherently experience tension. In previous theories, this tension was referred to as “string tension”, describing the intrinsic force in tip-suspended ultrafine nanowires attempting to contract. It has been demonstrated that the local minimum string tension, rather than the local minimum binding energy, is crucial for the stable existence of NWs [50]. Therefore, it is essential to assess the stability of Au NWs in terms of string tension. To more accurately simulate the real-world scenario of stretching a finite wire between two electrodes, we further derived the string tension of Au nanowires with varying $R_{e}$ values based on the binding energy. The string tension (*f*) of each structure was calculated according to the formula:

(2) $$f=\left(F-\mu N\right)/L$$

where *F* is the total wire free energy, *N* is the number of atoms, *L* is the length of the unit cell, and its value represents whether the NWs are in a compressed or stretched state, and *μ* is the chemical potential of gold (−3.15 eV) [51]. The variations in string tension as a function of the longitudinal dimension of Au NWs with diverse cross-sectional geometries, under both compressive and tensile loading conditions, are presented in Figure S1. The lowest point on the string tension–length curve for each structure corresponds to its most stable state, with the value at this point representing the string tension in the steady state. The string tension values for each structure are summarized in Figure 4b.

A clear monotonic increasing trend in string tension is observed across all T, R, and P series as a function of an increasing $R_{e}$. Among the three series of cross-sectional configurations, the string tension of the R series is generally lower than that of the others for a similar $R_{e}$, except for the structures with a radius around 3.67 Å ($R_{4}$) and 4.18 Å ($R_{5}$). The lowest string tension value in the R series indicates that it has the most stable structure among the three series. This phenomenon can be rationalized by the system’s maximal structural symmetry in the cross-sectional plane, which exhibits a close geometric correspondence to a hexagonal prismatic configuration, consistent with previously established theoretical predictions [27]. However, the calculated string tensions for $R_{4}$ and $R_{5}$ are found to be the highest compared to the T and P series when the $R_{e}$ is the same. This may be due to the surface composition of the cross-sectional structure. Geometrically, the cross-section of orientation Au NWs consists of (100) and (111) facets, with the (111) facet being the close-packed facet and exhibiting higher stability than the others [52]. For such ultrathin NWs, the surface effect may significantly influence the mechanical properties of the NWs, due to the large proportion of surface atoms [10]. Therefore, the proportion of (111) surface facets may impact the stability of orientation Au NWs. To systematically explore the relation between the proportion of (111) facets and the structural stability, we calculated the (111) facet proportion for each structure, as shown in Figure S2. The two highly symmetric structures, $R_{4}$ and $R_{5}$, exhibited lower (111) surface proportions and higher string tension, suggesting that a larger proportion of (111) facets correlates with greater structural stability. Our results indicate that, in addition to structural symmetry, the proportion of (111) surfaces is another key factor influencing the stability of Au NWs. This factor, previously overlooked, plays a crucial role in determining the structural stability of sub-3nm Au NWs.

**Figure 5:**
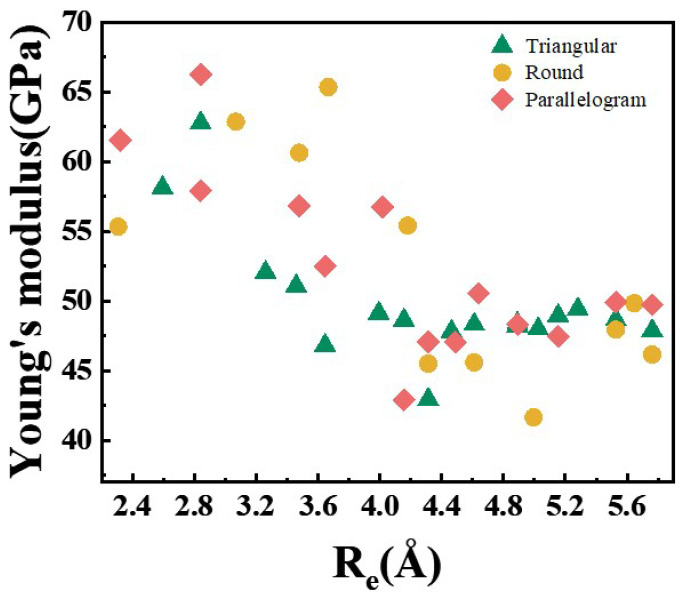
Calculated Young’s modulus versus the equivalent radius of Au NWs with different cross-sectional shapes.

The Young’s modulus of Au nanowires (NWs) with three distinct structural series was systematically calculated to elucidate their structural dependence, as presented in Figure 5. The Young’s modulus is calculated by using:

(3) $$Y=K\times L/\pi R_{e}^{2}$$

where *K* is the second derivative of binding energy when the structure is stabilized. Here, in order to better calculate the *K* value, we fitted the DFT calculated binding energy with the universal binding energy curves [53,54]:

(4) $$E_{binding}=-a(x-x_{0})e^{-b(x-x_{0})}$$

The second derivative of the curve after fitting is obtained as the *K* value. It is observed that the Young’s modulus of Au NWs is correlated with the value of $R_{e}$. For thinner nanowires (with $R_{e}$ < 4.3 Å) values, the Young’s modulus of the R series is the highest, that of the P series is intermediate, and the T series exhibits the lowest modulus. This suggests that the Young’s modulus exhibits a dependence on structural symmetry, demonstrating a decreasing trend with increasing values of $R_{e}$. However, for thicker nanowires (with $R_{e}$ > 4.3 Å), the Young’s modulus becomes independent of the nanowire size, with its value fluctuating around 47.6 GPa. Through a systematic comparison of the results presented in Figure 5 and Figure S2, no correlation is observed between the proportion of the (111) surface and the Young’s modulus of <110> oriented Au NWs. This phenomenon can be attributed to the high fluidity of both the (100) and (111) surfaces in ultrathin Au NWs. Previous studies have suggested that the surface tension of nanowires plays a significant role in their mechanical properties [34,55]. Since the NWs with a smaller radius in Figure 3b have a higher number of surface atoms, their impact on the mechanical properties may be more significant.

**Figure 6:**
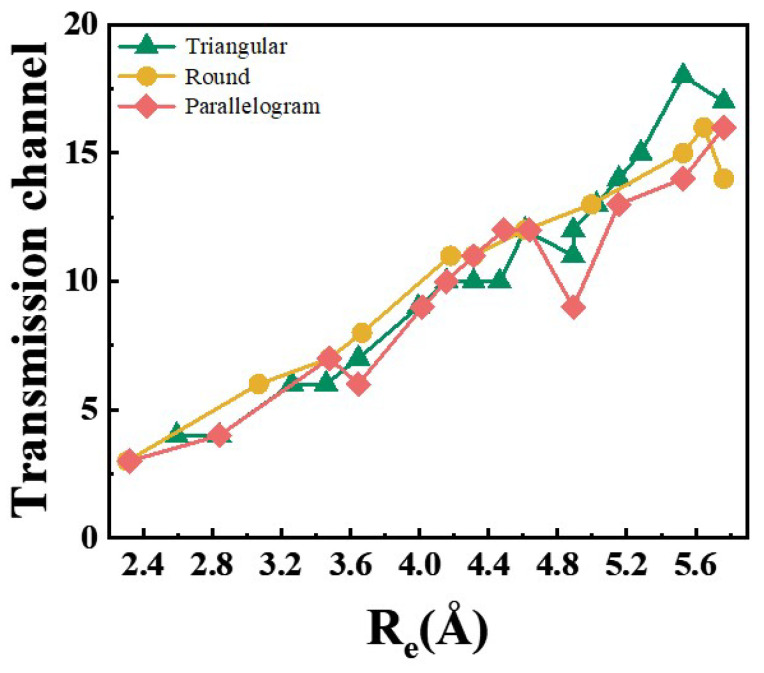
The calculated conductive channel numbers of T, R, P series.

The electrical conductivity of ultrathin NWs exhibits a direct correlation with the cross-sectional area of their corresponding atomic structure. In experimental investigations, the atomic configuration of NWs is typically deduced through the systematic assessment of their cross-sectional dimensions based on precise conductance measurements. Consequently, accurate conductivity characterization serves as a critical parameter for both determining the precise atomic arrangements of NWs and facilitating their potential applications in nano-electronic devices [47,48]. Therefore, we predict the conductivity values of various structures using DFT calculations to help experimental scientists more effectively validate their experimental results. In terms of electrical properties, it has been reported that the number of conductive channels is determined by the number of bands crossing the Fermi level, according to the Landauer formula [56]. Here, the relationship between the conductive channel numbers and the $R_{e}$ of the T, R, and P series is discussed to study their electrical properties and is shown in Figure 6 (all the band structures of the T, R, P series are shown in Figure S3). The number of conductance transport channels in each series shows an increasing trend with the increase in the $R_{e}$, which is similar to the results of previous studies [57], and the structure with the $R_{e}$ of 5.53 Å corresponds to the largest number of conductive channels, which is 18. Our calculations reveal that the number of conductive channels in the nanowires increases from 4 to 18 as the $R_{e}$ rises from 2.31 Å to 5.76 Å. Furthermore, when the $R_{e}$ is less than 4.2 Å, the R series structure with the highest symmetry exhibits the most conductive channels among the three series for a given $R_{e}$. This indicates that NWs with a higher cross-sectional symmetry are more likely to possess more conductive pathways when the NWs’ size is small. However, this difference in conductance is not significant. For instance, the R8, R9, and R10 structures exhibit a greater number of transmission channels, with values of 13, 15, and 16, respectively. In addition, the predicted conductivity values of the structures shown in Figure 2 are consistent with the calculated conductivity values.

**Figure 7:**
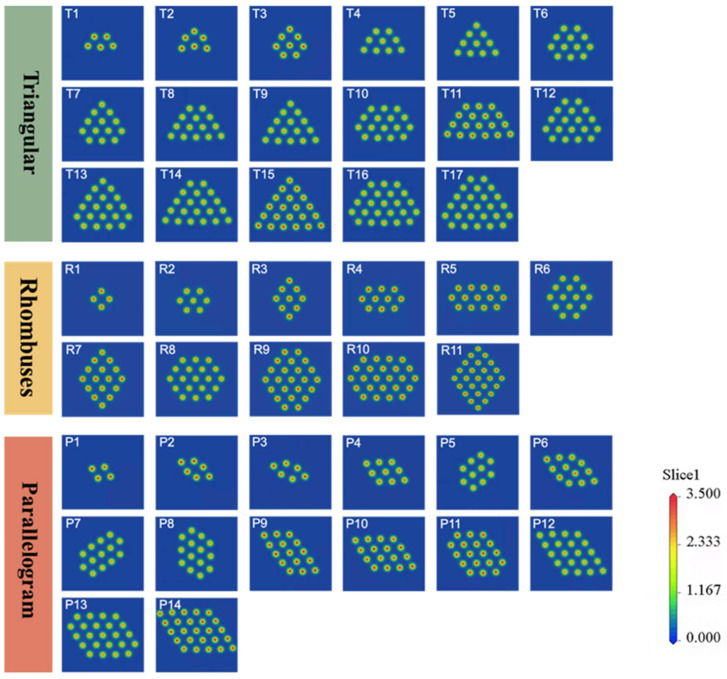
The calculated charge density contour of Au NWs for T, R, P series.

To better characterize the electrical properties of Au NWs with T, R, and P series cross-sections, we examined the charge density contours of Au NWs with different structures, as shown in Figure 7. The metallic behavior of the Au NWs is evidenced by the charge contours. Au NWs with T, R, and P series cross-sections all exhibit enhanced interfaces with metallic bonding features, as described by Silva et al. [58] and Liang et al. [59].

## 4. Conclusions

In summary, the mechanics and electrical property of sub-2nm Au NWs with various cross-sectional structures were investigated through the in situ TEM technique and DFT simulations from three perspectives: structural stability, Young’s modulus, and electrical conductivity. It is found that, in addition to the structural symmetry of the cross-section, the proportion of (111) densely packed surfaces in the cross-section is a critical factor influencing the structural stability of ultrathin Au NWs, a consideration that has been overlooked in previous studies. Furthermore, the Young’s modulus of Au NWs exhibits distinct characteristics, with the $R_{e}$ equal to three acting as a boundary. Specifically, when the $R_{e}$ is less than 4.3 Å, the Young’s modulus is influenced by the symmetry of the cross-sectional structure and decreases as the radius increases. However, when the $R_{e}$ exceeds 4.3 Å, neither the cross-sectional symmetry nor the radius has any significant effect on the Young’s modulus. Moreover, the Young’s modulus of the Au NWs is unaffected by the proportion of densely packed (111) surfaces, regardless of their size. This suggests that, due to the high fluidity of both the (100) and (111) surfaces in Au NWs, their Young’s modulus remains independent of the surface configuration. Furthermore, the electronic properties of Au NWs were systematically investigated through comprehensive analysis of both the density of conducting channels and the spatial charge distribution. The electrical conductivity of Au NWs, which exhibits a strong correlation with their cross-sectional dimensions and serves as a critical indicator for structural characterization, was quantitatively evaluated using first-principles band structure calculations. The results show that Au NWs, which exhibit high cross-sectional symmetry, are likely to have a greater number of conductive pathways for a given value of $R_{e}$. Our results shed light on the factors that influence the structural stability, Young’s modulus, and electrical properties of ultrathin Au NWs, providing a foundation for their potential applications in nanodevices.

## Figures and Tables

**Table 1:** Energy, Mechanical, and Electrical Calculations Related to Stability of T Series, R Series and P Series Structures.

Configurations		N_atoms_	NG_0_	*E* (eV/atom)	*f* (ev/Å)	Proportion of (111) Facet	*Y*(Gpa)
**T series**	**T1**	5	4	−2.27	1.57	0.36	58.14
	**T2**	6	4	−2.39	1.63	0.63	62.77
	**T3**	8	6	−2.52	1.78	0.84	52.07
	**T4**	9	6	−2.53	1.99	0.46	51.09
	**T5**	10	7	−2.58	2.02	0.63	46.83
	**T6**	12	9	−2.64	2.17	0.63	49.13
	**T7**	13	10	−2.67	2.20	0.77	48.64
	**T8**	14	10	−2.67	2.37	0.51	42.95
	**T9**	15	10	−2.70	2.40	0.63	47.82
	**T10**	16	12	−2.70	2.52	0.51	48.32
	**T11**	18	11	−2.72	2.72	0.42	48.40
	**T12**	18	12	−2.74	2.57	0.63	48.20
	**T13**	19	13	−2.76	2.61	0.74	48.00
	**T14**	20	14	−2.76	2.76	0.53	48.96
	**T15**	21	15	−2.77	2.80	0.63	49.43
	**T16**	23	18	−2.79	2.89	0.53	48.66
	**T17**	25	17	−2.81	2.96	0.63	47.88
**R series**	**R1**	4	3	−2.25	1.29	1	55.34
	**R2**	7	6	−2.44	1.75	0.63	62.86
	**R3**	9	7	−2.57	1.84	1	60.63
	**R4**	10	8	−2.55	2.12	0.46	65.35
	**R5**	13	11	−2.61	2.49	0.36	55.41
	**R6**	14	11	−2.69	2.26	0.77	45.51
	**R7**	16	12	−2.74	2.32	1	45.60
	**R8**	19	13	−2.76	2.58	0.63	41.65
	**R9**	23	15	−2.82	2.68	0.84	47.95
	**R10**	24	16	−2.81	2.86	0.53	49.84
	**R11**	25	14	−2.84	2.75	1	46.16
**P series**	**P1**	4	3	−2.20	1.36	0.46	61.52
	**P2**	6	4	−2.41	1.58	0.63	57.91
	**P3**	6	4	−2.36	1.69	0.3	66.26
	**P4**	9	7	−2.53	1.98	0.46	56.82
	**P5**	10	6	−2.58	2.01	0.72	52.49
	**P6**	12	9	−2.63	2.20	0.56	56.74
	**P7**	13	10	−2.65	2.28	0.77	42.91
	**P8**	14	11	−2.67	2.37	0.63	47.05
	**P9**	15	12	−2.68	2.48	0.56	47.05
	**P10**	16	12	−2.70	2.56	0.46	50.55
	**P11**	18	9	−2.74	2.58	0.63	48.33
	**P12**	20	13	−2.75	2.79	0.53	47.48
	**P13**	23	14	−2.79	2.88	0.53	49.91
	**P14**	25	16	−2.80	3.09	0.46	49.73

N_atoms_ represents the number of atoms in the cross-section, NG_0_ represents the number of conductive channel, *E* represents the binding energy, *f* represents the string tension, and *Y* represents the Young’s modulus.

## Data Availability

The data that support the findings of this study are available from the corresponding author upon reasonable request.

## Supplementary Materials

The following supporting information can be downloaded at: https://www.mdpi.com/article/10.3390/nano15060428/s1.
