# Supplementary material for: Ultrathin Gold Nanowires
Source: Nanomaterials (Basel). 2025 Mar 11;15(6):428. doi: 10.3390/nano15060428 (PMC11944545; doi:10.3390/nano15060428)
Supplement: Supplementary file 1 [file nanomaterials-15-00428-s001.zip › nanomaterials-3493496-supplementary.pdf]

Supporting Information

Ultra-thin gold nanowires

Shuo Liu,[a, ‡] Chunmeng Liu,[a,c, ‡] Ye Wang,[d,*] Jiaqi Zhang,[a,b,*] Shaobo Cheng,[a,b,*] Chongxin Shan,[a,b,]

a. Henan Key Laboratory of Diamond Optoelectronic Materials and Devices, Key Laboratory of Materials Physics, Ministry of Education, and School of Physics and Laboratory of Zhongyuan Light, Zhengzhou University, Zhengzhou 450052, China.
b. Institute of Quantum Materials and Physics, Henan Academy of Sciences, Zhengzhou 450046, China.
c. Center of Advanced Analysis & Gene Sequencing, Zhengzhou University, Zhengzhou 450001, China.
d. Key Laboratory of Material Physics, Ministry of Education, School of Physics and Microelectronics, Zhengzhou University, Zhengzhou 450052, China.

Supporting Figures

(a)

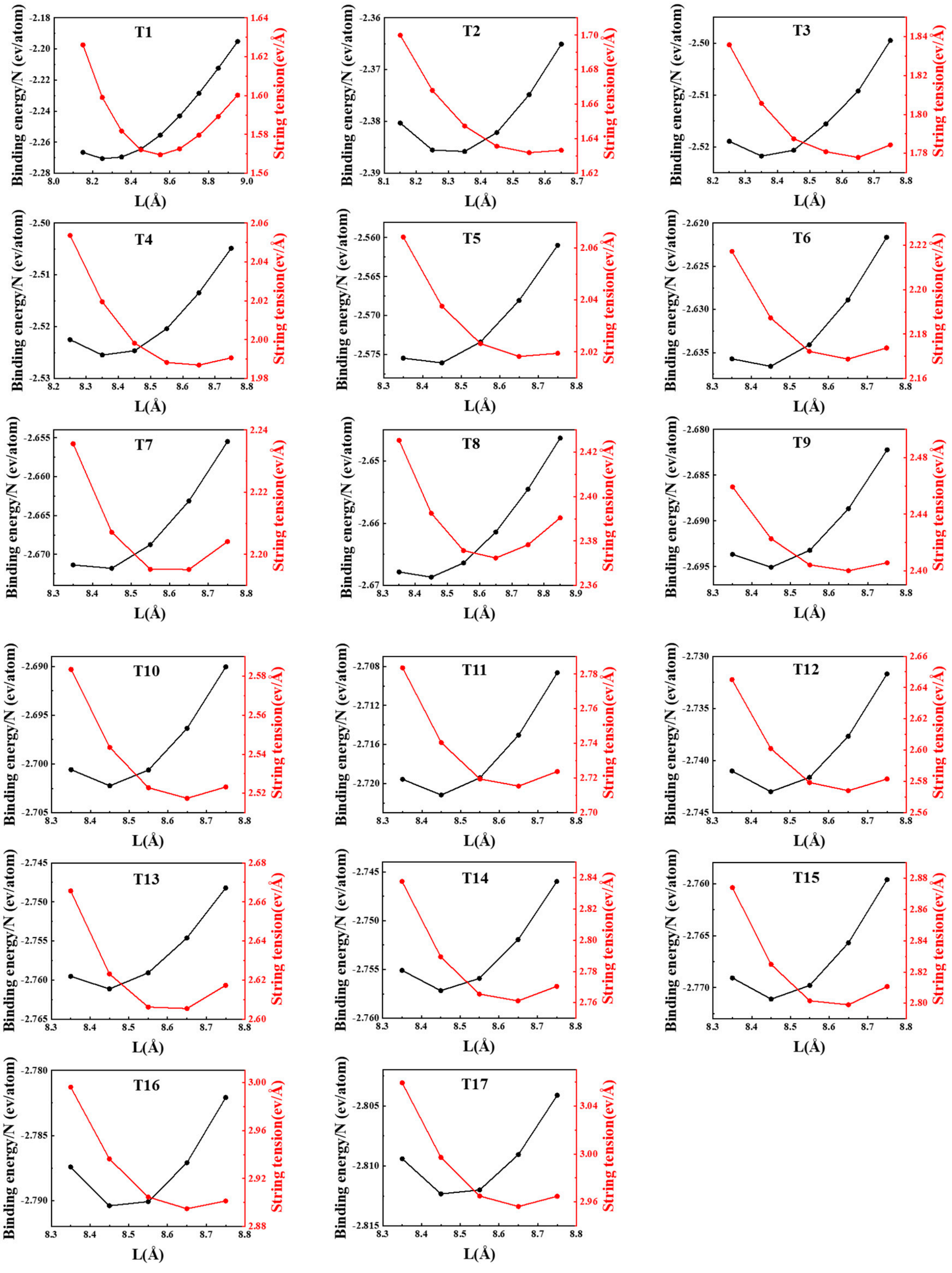

(b)

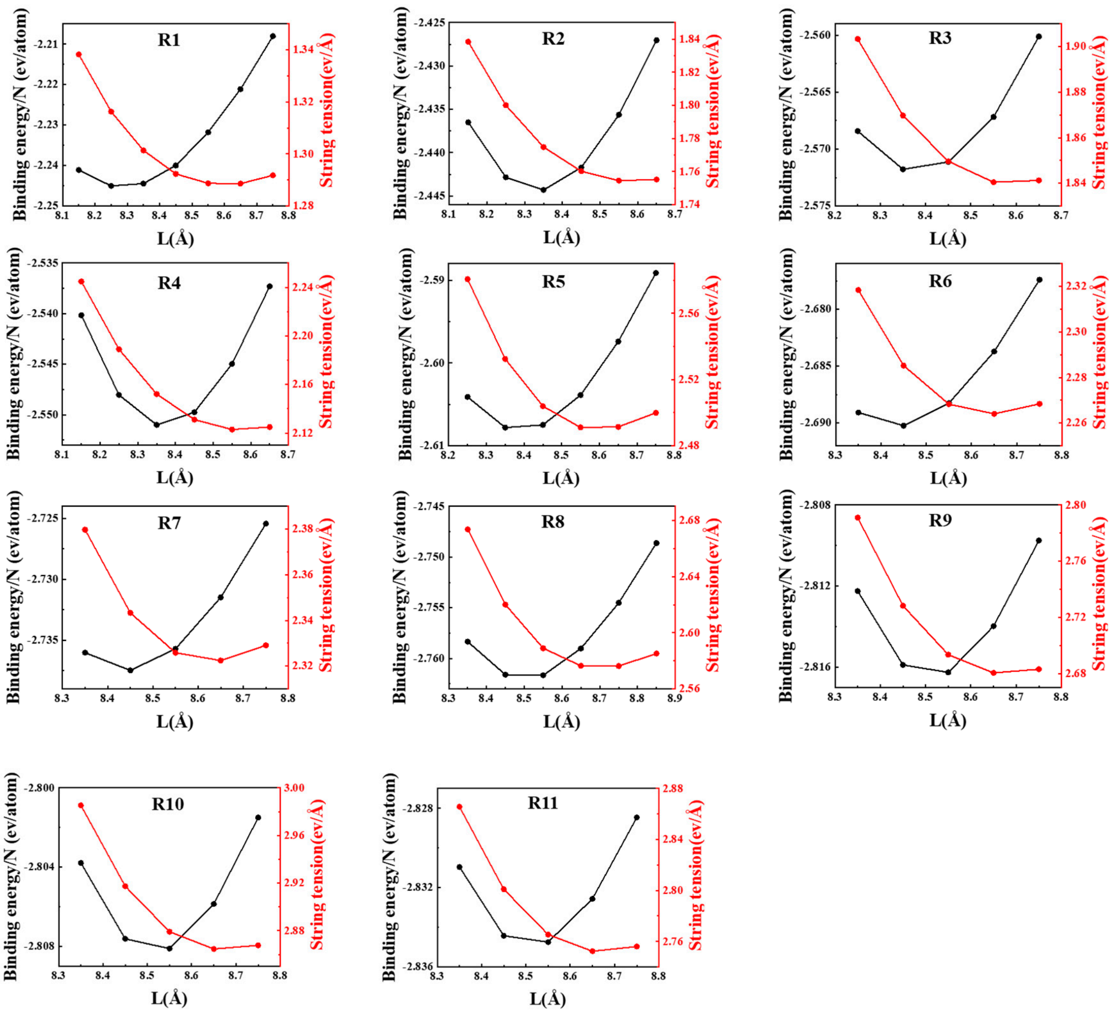
R1
R2
R3
R4
R5
R6
R7
R8
R9
R10
R11
Binding energy/N (ev/atom)
String tension(ev/Å)
L(Å)

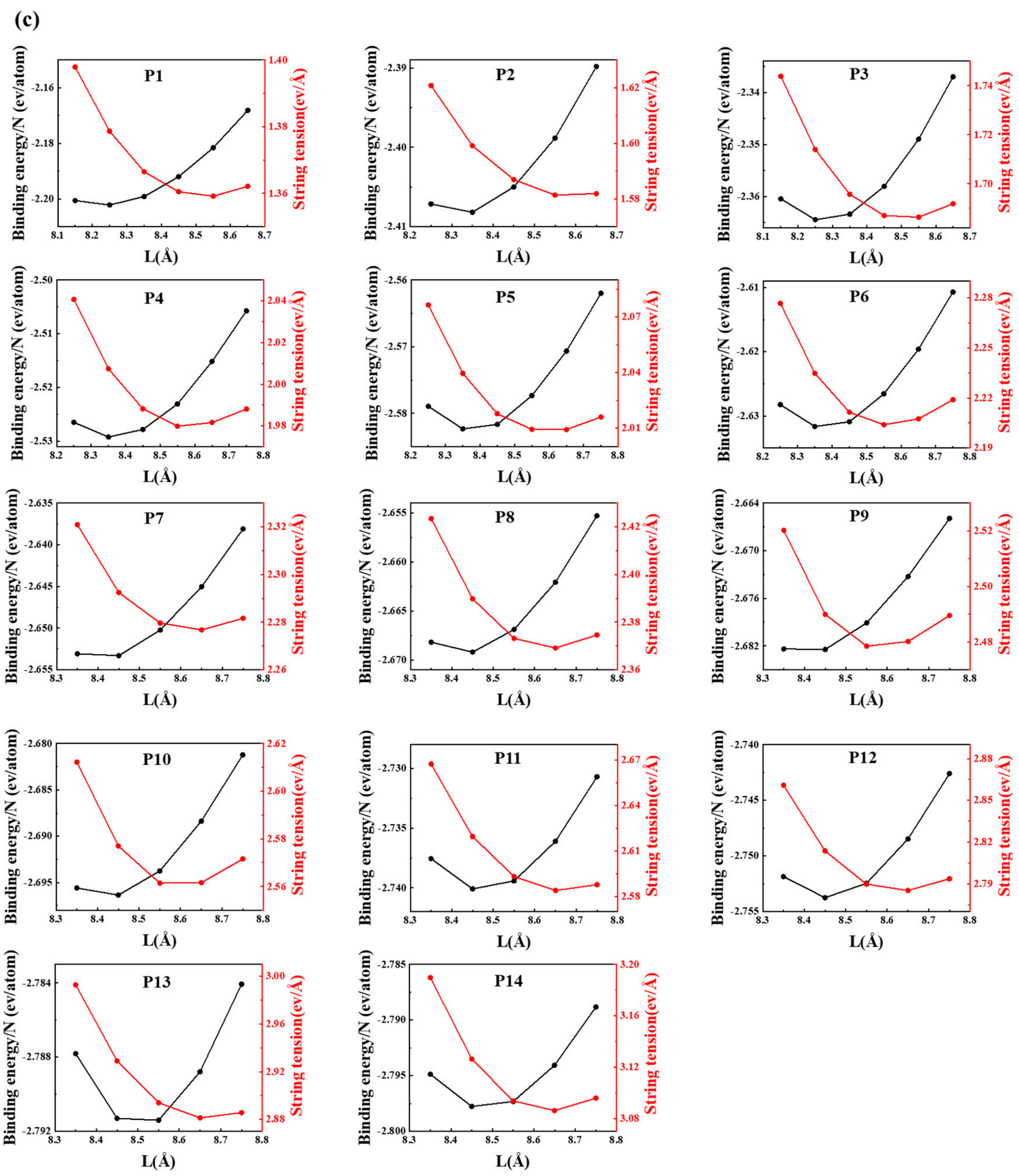

Figure S1. Calculated binding energy/N (left) and string tension/N (right) for Au NWs with different lengths (L) and cross-sectional structures: (a) T series, (b) R series, and (c) P series.

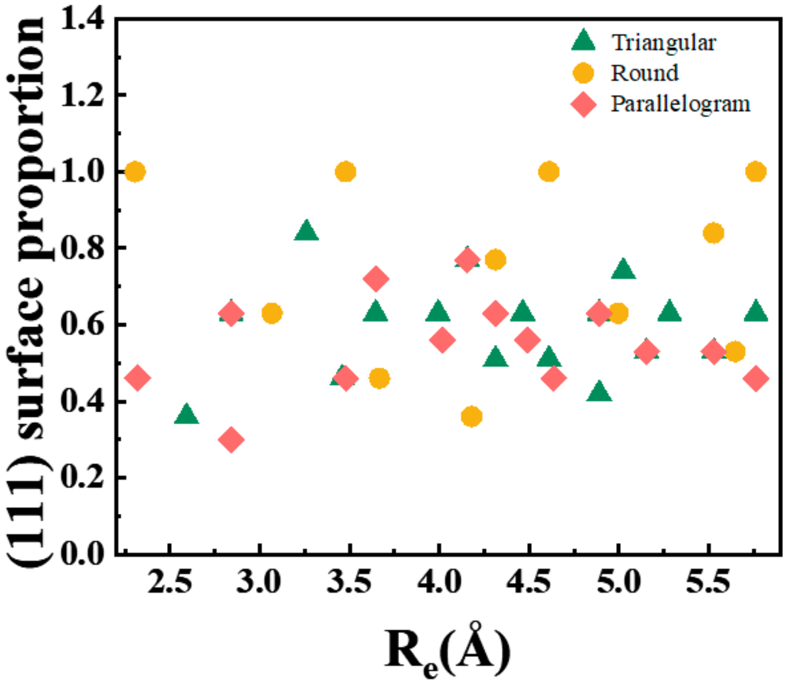

Figure S2. The proportion of (111) surface for the T, R, and P series.

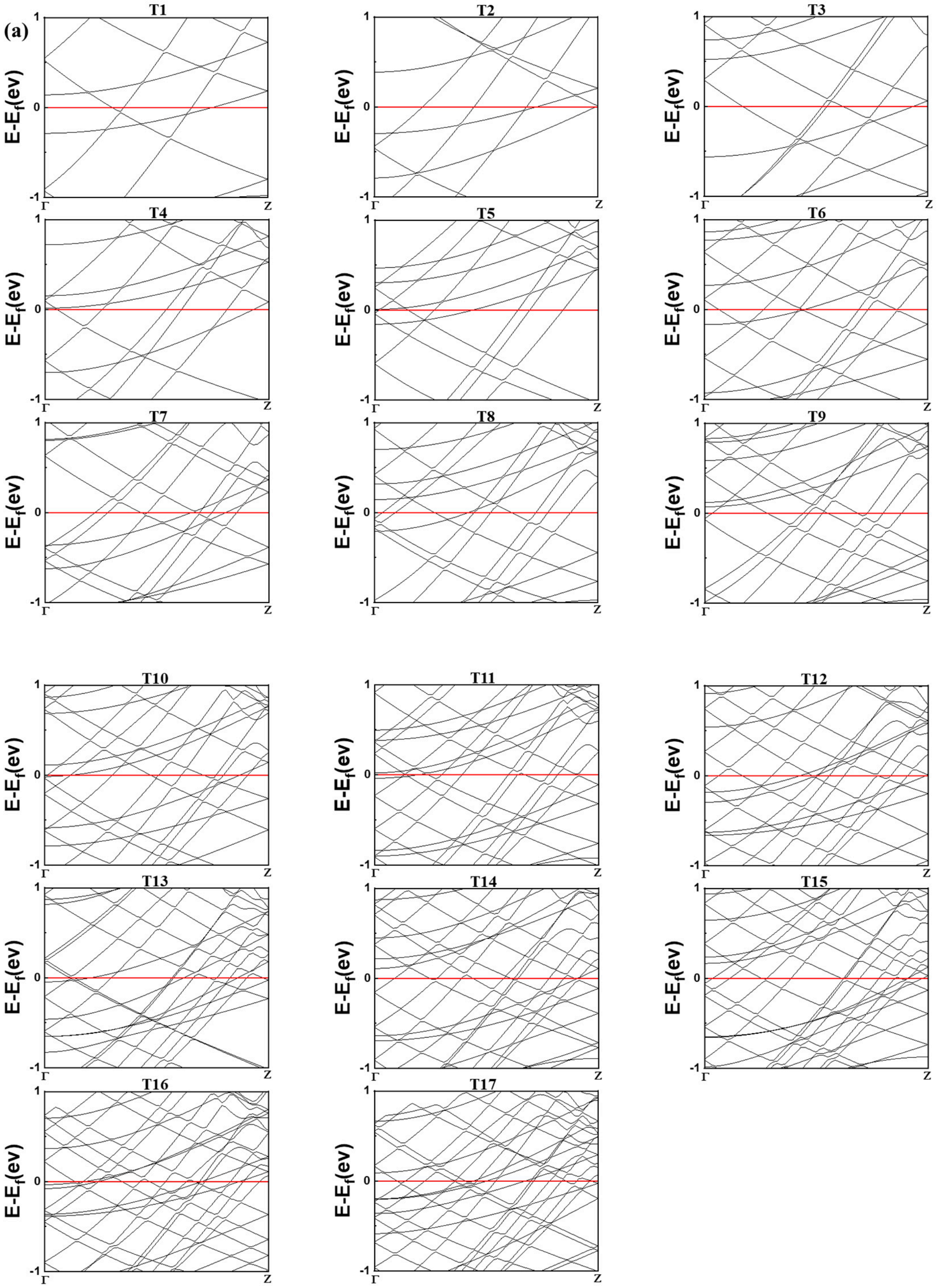
(a)
T1
T2
T3
T4
T5
T6
T7
T8
T9
T10
T11
T12
T13
T14
T15
T16
T17
E-Ef(ev)
1
0
-1
Γ
Z

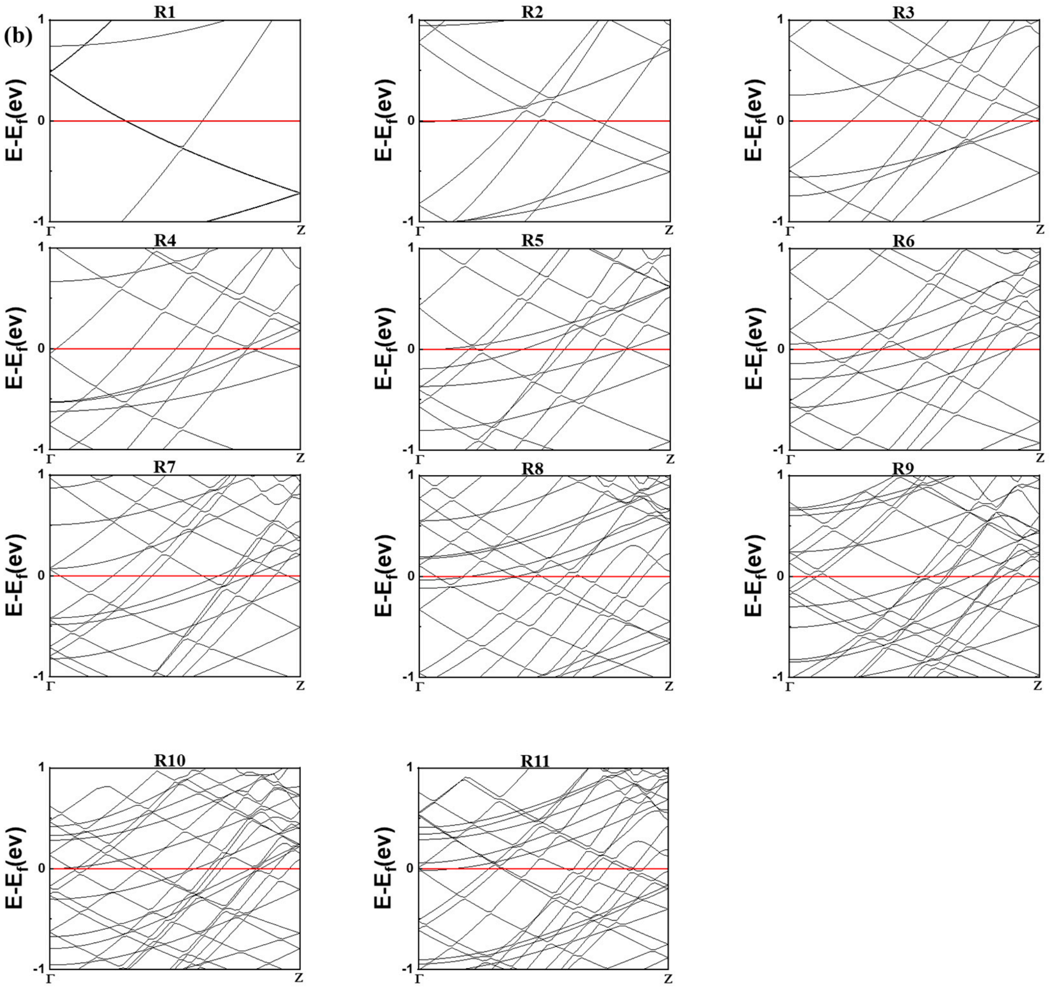
(b)
R1
R2
R3
R4
R5
R6
R7
R8
R9
R10
R11
E-E$_f$(ev)
1
0
-1
Γ
Z

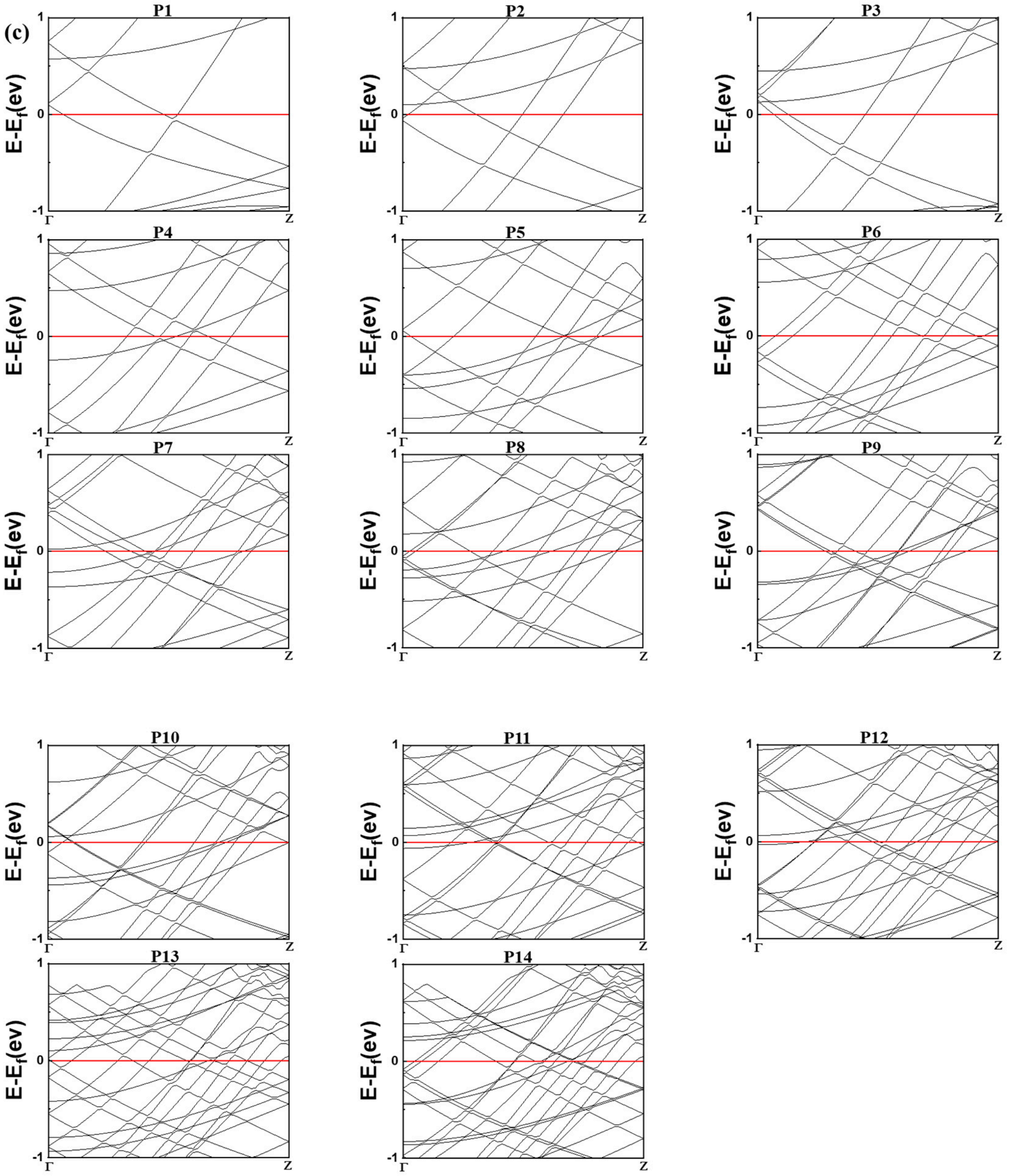

Figure S3. Calculated band structures of Au NWs cross-sections with different lengths (L) for the (a) T, (b) R, and (c) P series, respectively.
